# Cathepsin B induces kidney diseases through different types of programmed cell death

**DOI:** 10.3389/fimmu.2025.1535313

**Published:** 2025-03-10

**Authors:** Yunlong Zhao, Yong Zhuang, Jie Shi, Haojun Fan, Qi Lv, Xiaoqin Guo

**Affiliations:** ^1^ Institute of Disaster and Emergency Medicine, Tianjin University, Tianjin, China; ^2^ Key Laboratory for Disaster Medicine Technology, Tianjin University, Tianjin, China; ^3^ Wenzhou Safety (Emergency) Institute, Tianjin University, Wenzhou, China

**Keywords:** cathepsin B, kidney disease, programmed cell death, mechanism, therapeutic target

## Abstract

Cathepsin B (CTSB), a key cysteine protease, plays essential roles in physiological and pathological processes. As research progresses, interest in how CTSB triggers different types of programmed cell death (PCD) to induce the onset and development of diseases is increasing. Several recent studies suggest that different types of PCD mediated by CTSB play key roles in kidney diseases. In this review, we outline the fundamental mechanisms by which CTSB triggers different types of PCD in several kidney diseases and discuss the function of CTSB in various segments of the kidney. Moreover, we explore the possibilities and prospects of using CTSB as a therapeutic target for kidney diseases.

## 1 Introduction

CTSB is a cysteine protease of the papain family that is widely expressed in human tissues and is localized mainly in lysosomal compartments and subcellular endosomes (1). Compared with other cathepsins (cathepsins A, C, D, E, F, G, H, J, K, L, O, S, T, V, W, Y, and Z), CTSB has received intense attention from researchers because of its involvement in a myriad of physiological and pathological processes within the human body (2).

Since the discovery of CTSB in the 1950s, scholars have explored many of its functions. Protein synthesis and degradation are dynamic equilibrium processes. CTSB plays a crucial role in the intracellular degradation and processing of proteins and ensures this equilibrium through its proteolytic activity (1). CTSB not only is involved in the regular turnover of proteins but also participates in the activation of bioactive substances. CTSB can mediate different types of PCD (3, 4). For example, during the process of cell apoptosis, CTSB can cleave specific substrates, thereby promoting the process of cell apoptosis. In autophagic cell death, CTSB participates in the formation of autophagosomes through its proteolytic activity, ensuring the effective progression of autophagy and thus affecting the fate of cells (1). In addition, CTSB participates in the onset and progression of inflammatory reactions by regulating the release of inflammatory mediators, cell signaling, and other pathways.

CTSB is closely related to various diseases. In cancer, CTSB promotes the invasion and metastasis of tumour cells and affects the development of cancer by regulating the tumour microenvironment (5). In rheumatoid arthritis and osteoarthritis, the increased activity of CTSB leads to the destruction of joint tissue (6). In osteoporosis, CTSB participates in the process of bone resorption and affects bone density (7). In addition, CTSB has been shown to play a pathological role in diseases such as intracerebral hemorrhage, liver fibrosis, pancreatitis, Alzheimer’s disease, and inflammatory respiratory diseases (8–10).

CTSB, a key mediator, has been shown to play an important role in kidney disease. An increasing body of evidence suggests that CTSB induces the occurrence of kidney diseases through multiple cell death pathways (3). Here, we focus on elucidating the mechanisms by which CTSB induces kidney disease through different types of PCD pathways. We also review the process of CTSB maturation, its physiological functions, and the roles it plays in different segments of the kidney. In addition, the CTSB protein has attracted increasing attention as a therapeutic target for diseases (11, 12), and we further explore the role of CTSB in the treatment of kidney diseases.

## 2 The maturation and physiological functions of CTSB

The process of CTSB maturation involves a series of finely regulated steps. First, CTSB is synthesized on ribosomes as the inactive form preprocathepsin B. This preprocathepsin carries a signal peptide that ensures that it is properly directed to the endoplasmic reticulum. In the endoplasmic reticulum, signal peptides are cleaved and the precursor is converted into procatheapsin B. Procathepsin B subsequently undergoes glycosylation in the Golgi apparatus, where it is modified with sugar chains with phosphorylated mannose. These sugar chains allow procathepsin B to be recognized by the mannose 6-phosphate receptor, directing it towards the lysosome for translocation (3). In the acidic environment of the lysosome, precursor enzymes remove precursor peptides through autocatalysis or the action of other proteases, ultimately forming mature CTSB with activity. Mature CTSB is composed of a heavy chain and a light chain, which are tightly connected through disulfide bonds (13). This maturation process ensures that CTSB can achieve its highest efficacy in its operating environment, which is crucial for its activity and functionality. Within the cell, CTSB activity is strictly regulated to ensure that it performs its physiological functions at the right time and in specific locations. Furthermore, CTSB demonstrates enzymatic activity across a broad pH range, functions effectively from pH 3.0 to 7.0, and may suffer irreversible inactivation in overly alkaline environments.

Although CTSB often plays a pathological role in diseases such as cancer and neurodegeneration, it also has important physiological functions in normal cells. Recent research has revealed the decisive impact of the tissue and cell specificity or subcellular localization of CTSB on the diversity of its physiological functions (14). One of the main physiological functions of CTSB is to participate in antigen processing and presentation. Dendritic cells are key to initiating immune responses; they rely on CTSB to degrade antigens and then present them to T cells. The activation of B cells and CD8+ T cells is significantly facilitated by this process, underscoring the pivotal role of CTSB in the adaptive immune response (15, 16). In addition to its role in antigen processing and presentation, CTSB is involved in regulating cellular redox homeostasis. Various redox molecules can reversibly modify CTSB, affecting redox homeostasis in mammalian cells and tissues. In addition, CTSB is related to the regulation of cellular ageing. By affecting the expression of various receptors and ligands associated with ageing, CTSB might be involved in regulating the fate of senescent cells (17). Overall, CTSB is a multifunctional enzyme that plays a variety of physiological roles in mammalian cells and tissues, from antigen processing to the regulation of redox balance and the regulation of cellular ageing. CTSB is involved in numerous physiological processes that are crucial for normal cellular function.

## 3 Function of CTSB in the kidney

**Figure 1:**
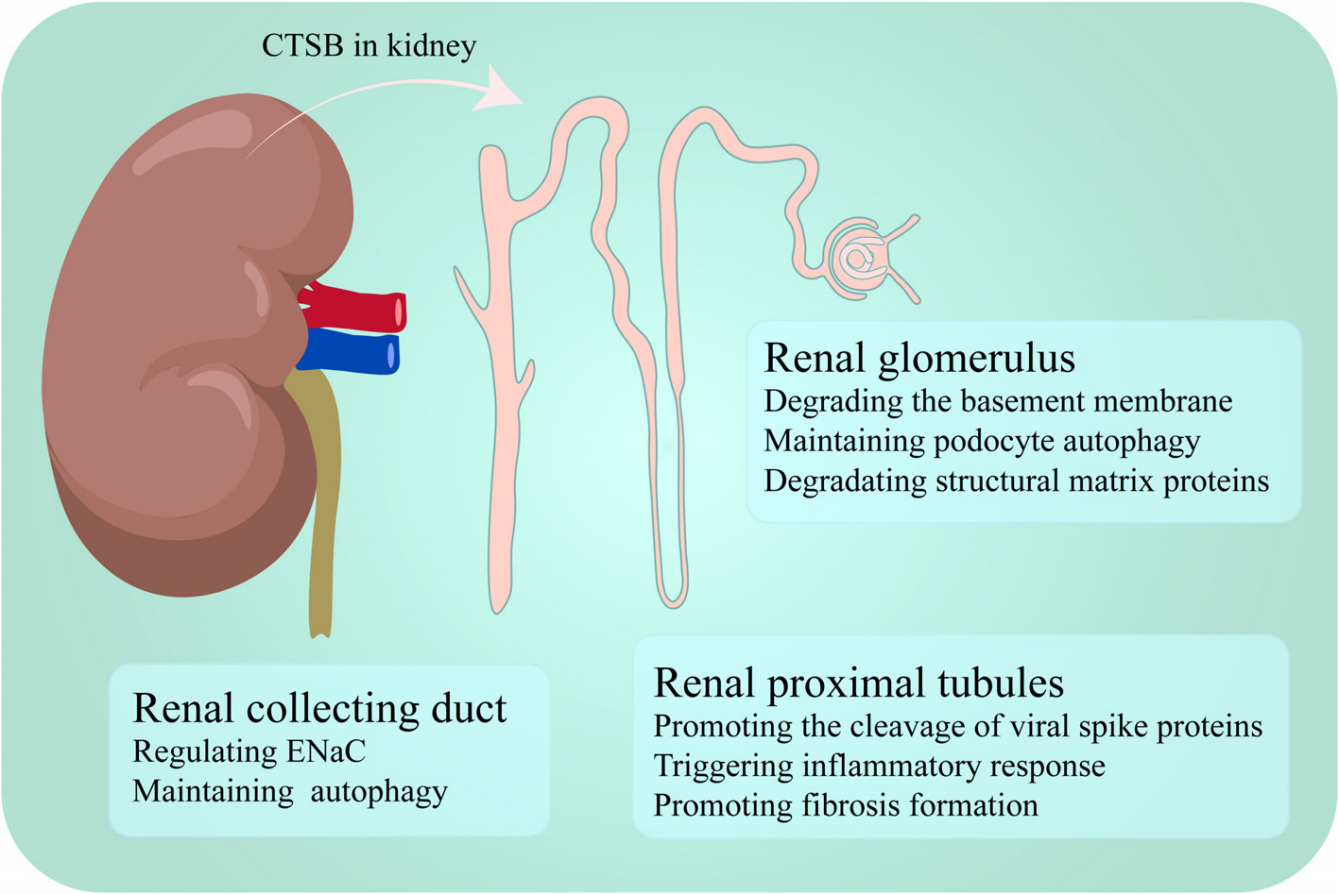
CTSB performs different functions in different segments of the kidney.

CTSB is widely present in the cortex and medulla of the kidney and is expressed in various kidney cells, with particularly high expression levels in endothelial cells and proximal tubules (18–20). CTSB plays different roles in different segments. In this section, the different or similar roles of CTSB in the glomerulus, tubules are described (**Figure 1**).

### 3.1 Function of CTSB in the renal glomerulus

The glomerulus is a key structure involved in kidney disease, and the impairment of its function not only leads to a decrease in the kidney’s filtration capacity but also increases the accumulation of toxins in the body, affecting overall health (21). In a spatial proteomics and immunological characterization study of kidney samples from patients with lupus nephritis, unique gene expression patterns were observed in renal areas with different functions, including CTSB, which presented significantly increased expression in the glomerulus, along with four other genes. Although the specific mechanism of action of CTSB in the glomeruli of patients with lupus nephritis has not been fully elucidated, this finding highlights its potential importance in glomerular function and disease progression (22). In contrast to the above trend of CTSB expression, in the streptozotocin-induced diabetes rat model and the high glucose-treated glomerular mesangial cell model, the expression of cathepsins, including CTSB, and other degrading enzymes decreased. Under physiological conditions, CTSB is involved in the degradation of various proteins in the glomerulus, such as laminin and collagen (23). CTSB has also been shown to degrade the basement membrane of the glomerulus (23, 24). In diabetic nephropathy, the weakening of CTSB activity leads to reduced degradation of the basement membrane, resulting in excessive deposition of extracellular matrix components, further exacerbating the thickening of the glomerular basement membrane, which leads to glomerular filtration dysfunction and inflammatory responses, thereby indirectly worsening diabetic nephropathy (23–26). In addition, the podocytes lining the exterior of the glomerular basement membrane are crucial for preserving the kidney’s filtration barrier (27). In diabetic patients, podocytes often experience lysosomal dysfunction, and the activity of lysosomal enzymes, including CTSB, decreases after treatment with advanced glycation end products. When resveratrol and vitamin E are administered to podocytes treated with advanced glycation end products, CTSB enzyme activity increases, and podocyte damage is reduced (28, 29). Reactive oxygen species (ROS) are mediators of apoptosis induced by advanced glycation end products, and autophagy may be triggered to repair damage caused by ROS, thereby promoting the survival of cells treated with advanced glycation end products (30, 31). Therefore, the activation of podocyte autophagy during lysosomal recovery may play a protective role in diabetic nephropathy (29). However, in mouse models of glomerular or podocyte injury induced by nephrotoxic serum, CTSB has been identified as a key mediator of glomerular injury. Mice with CTSB knockout exhibited greater tolerance and faster recovery under the induction of nephrotoxic serum (32).

### 3.2 Function of CTSB in renal tubules

CTSB has received the most attention in the study of renal tubules, as it is involved in the occurrence of various kidney diseases in renal tubules. CTSB mainly exerts its effects in the proximal tubules and collecting duct of the renal tubule.

#### 3.2.1 Function of CTSB in the renal proximal tubules

CTSB also participates in regulating autophagy in the proximal tubules. Receptor-interacting protein kinase 3 (RIPK3) damages lysosomes in sepsis-induced acute kidney injury (S-AKI). When RIPK3 inhibitors are used to treat mouse renal tubular cells, CTSB activity resumes and S-AKI is relieved (33). Circulating CTSB may be filtered by the glomerulus and taken up by megalin in proximal tubular cells to support lysosomal function (34). In a study of severe acute respiratory syndrome coronavirus 2 (SARS-CoV-2) tropism to the kidney, CTSB was identified as a key factor promoting the cleavage of the viral spike protein, a process that facilitates the invasion of SARS-CoV-2 into proximal tubule epithelial cells type 3 cells, which are a subtype of renal tubular cells. Therefore, most kidney damage caused by SARS-CoV-2 is often observed in the proximal tubular region (35, 36). In addition, CTSB is closely associated with the inflammatory response and fibrosis formation in the proximal tubules (37, 38).

#### 3.2.2 Function of CTSB in the renal collecting duct

Similarly, the CTSB has irreplaceable significance in the collecting duct. Like in the glomerulus and tubules, lysosomal CTSB can also participate in protein degradation in the collecting ducts, alleviating kidney disease by regulating autophagy. For example, polycystic kidney disease 1 (PKD1) gene deficiency often leads to polycystic kidney disease. In cells isolated from the inner medullary collecting ducts of mouse kidneys with PKD1 gene knockout, the absence of the PKD1 gene leads to lysosomal damage and reduced processing and activity of CTSB. This change occurs because PKD1 gene defects can cause an increase in calcium-dependent protease activity, which triggers the hydrolysis of lysosome-related membrane proteins and causes lysosome dysfunction. When treated with calcium-dependent protease inhibitors, lysosome-related functions are restored, and CTSB activity is also restored (39).

In the collecting duct, CTSB is also considered an important participant in sodium retention and hypertension. Myristoylated alanine-rich c-kinase substrate family proteins in the kidney are important for regulating epithelial sodium channel (ENaC) function and blood pressure in the renal cortical collecting ducts, and an upregulation of CTSB contributes to increased hydrolysis of myristoylated alanine-rich c-kinase substrate family proteins in diabetic kidneys (40, 41). Human ENaC is composed of four subunits: α, β, γ, and δ. Among them, α - ENaC is an essential component for the function of ENaC. CTSB can cleave α - ENaC and activate ENaC *in situ*, or it may increase the activity of ENaC by acting on proteins known to regulate the insertion, gating, recycling, or degradation of ENaC, thereby promoting hypertension in patients with nephrotic syndrome (42–44). In addition, when metformin is used to treat diabetes, it can alleviate the onset and development of diabetes-associated hypertension by targeting the CTSB-ENaC pathway in collecting duct cells (45).

## 4 CTSB induces kidney diseases through PCD

**Figure 2:**
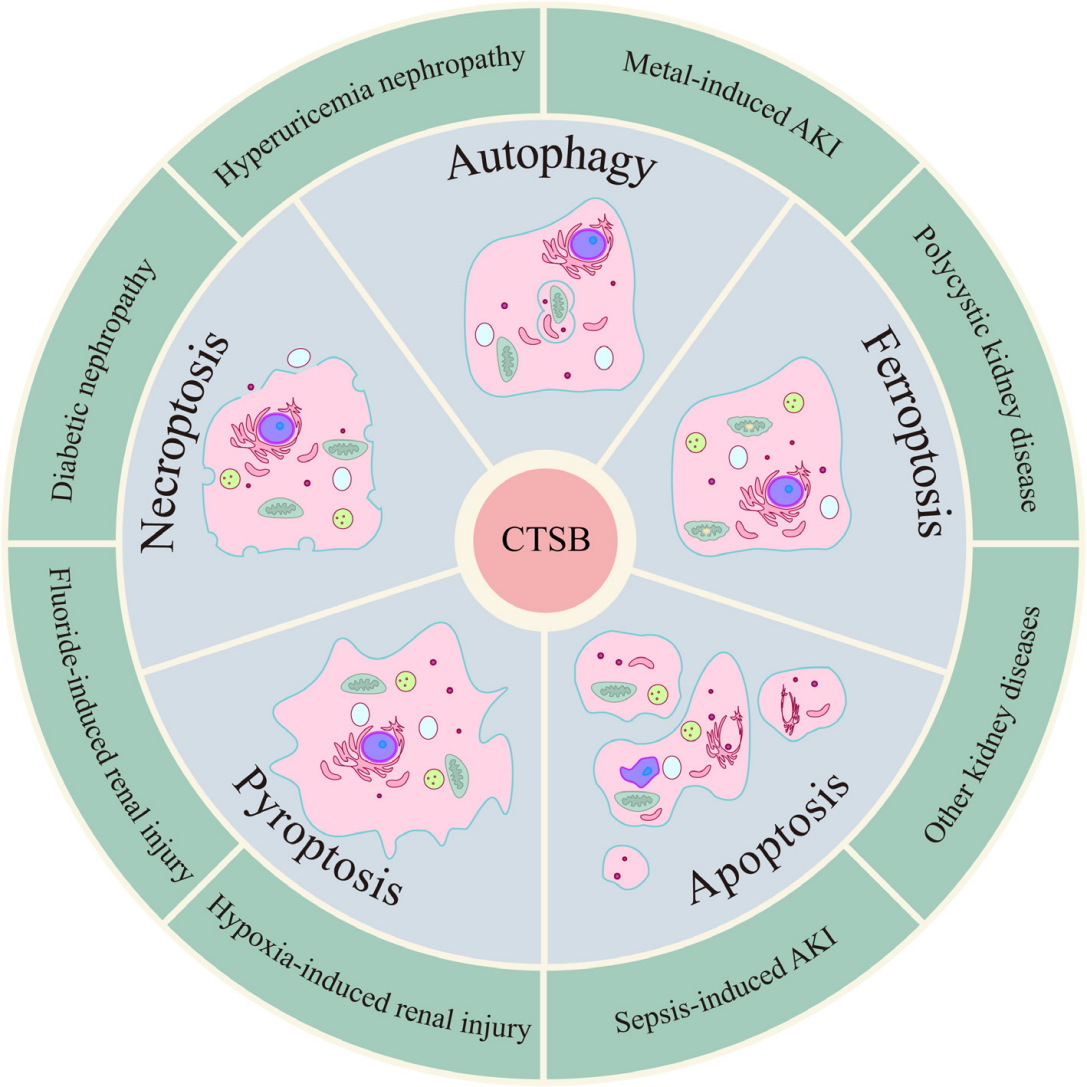
CTSB induces kidney diseases by mediating/regulating multiple PCD modalities.

CTSB has been shown to be significantly involved in the pathogenesis of various kidney diseases, such as acute kidney injury (S-AKI and metal-induced AKI), diabetic nephropathy, polycystic kidney disease, hyperuricemia nephropathy, kidney cancer, glomerulonephritis, and kidney transplantation (46–52). PCD is a type of cell death that mainly includes apoptosis, pyroptosis, necroptosis, autophagy, ferroptosis and cuproptosis and is instrumental in health and disease (53–55). Different types of PCD are intricately linked, and CTSB, as one of the common mediators of different types of PCD, plays a key role in a variety of kidney diseases (**Figure 2**).

### 4.1 CTSB induces kidney diseases by mediating apoptosis

The integrity of lysosomal membranes is crucial for determining cell fate. Under normal circumstances, lysosomal membranes are relatively stable and are not freely permeable. However, under certain physiological or pathological conditions, lysosomal membrane permeabilization (LMP) occurs. LMP refers to the disruption of lysosomal membrane integrity, leading to the release of its contents (such as hydrolases, etc.) into the cytoplasm. Under physiological conditions, LMP can promote the limited release of lysosomal enzymes, which play important roles in the cytoplasm and nucleus, supporting processes like cell division, cytoskeleton rearrangement, and epigenetic regulation (56–59). Under pathological conditions, LMP causes the massive release of lysosomal contents, thereby triggering large-scale hydrolysis of cytoplasmic contents, leading to fatal consequences for the cell (57). For example, proteinuria has been recognized as a biomarker for kidney disease, and proteinuria with nephrotoxicity can damage lysosomes in renal tubular epithelial cells and increase lysosomal membrane permeability (60, 61). When LMP occurs, a variety of hydrolytic enzymes, including CTSB, are released into the cytoplasm or extracellularly, which in turn triggers cell death (62, 63).

**Figure 3:**
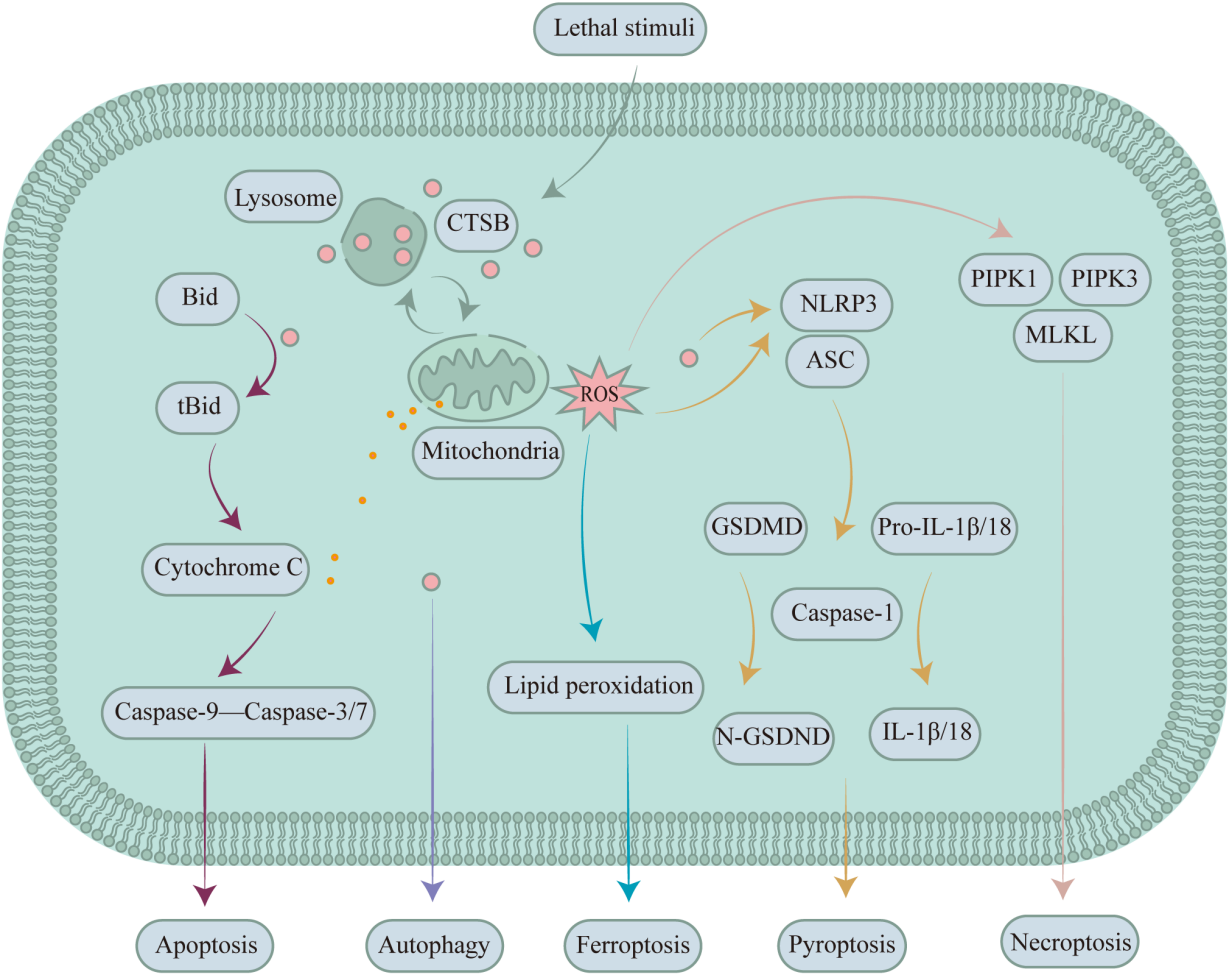
CTSB expressed in renal tubular epithelial cells participates in mediating/regulating different types of PCD in kidney diseases. During apoptosis, CTSB is involved in mediating renal apoptosis by cleaving Bid. During autophagy, CTSB serves as a key protease that regulates autophagy flux and thereby affects kidney diseases. In ferroptosis, CTSB mediates renal disease by inducing the accumulation of ROS and intelligent delivery systems that respond to CTSB activity via the peroxidation of polyunsaturated fatty acids. During pyroptosis, CTSB triggers NLRP3 to induce pyroptosis in the kidney. During necroptosis, CTSB facilitates the recruitment and activation of PIPK1, PIPK3, and MLKL by promoting the generation of ROS, thereby inducing necroptosis in the kidney.

Studies have shown that CTSB is involved in the initiation of apoptosis. The pathways through which CTSB initiates apoptosis mainly include caspase-dependent and caspase-independent pathways. Cruzipain, a cysteine protease closely related to human cathepsins, has been shown to directly activate caspase-3 and caspase-7, inducing cell apoptosis. CTSB primarily participates in the cellular apoptosis pathway by cleaving the proapoptotic factor BH3 interacting domain death agonist (Bid). When Bid is cleaved by CSTB, it forms an activated form of the receptor called truncated BH3 interacting domain death agonist (tBid). tBid acts on mitochondria and is able to promote mitochondrial membrane permeabilization (MMP), resulting in the liberation of cytochrome C from the mitochondrial intermembrane space, thereby initiating the caspase cascade and triggering apoptosis (**Figure 3**) (64). LMP with CTSB leakage has been shown to be strongly associated with the development of S-AKI. In an *in vitro* S-AKI model induced by lipopolysaccharide treatment in the human renal proximal tubular epithelial cell line-2 (HK-2 cells), CTSB activity and mRNA expression are elevated, and CTSB leads to apoptosis through activation of the mitochondrial apoptotic pathway (65). Inhibition of CTSB activity inhibits apoptosis and increases cell viability. CA074 is a potent inhibitor of CTSB, and inhibition of CTSB activity by CA074 can effectively block the activation of the apoptosis-related markers BCL-2-associated X protein (BAX)/Caspase-3/poly ADP-ribose polymerase (PRAP) to reverse apoptosis in HK-2 cells (65). Metallic lead is toxic to the kidneys and causes damage to kidney tubular cells. When rat proximal tubular are exposed to lead, CTSB and cathepsin D (CTSD) translocate from lysosomes with LMP into the cytoplasm. The combined actions of CTSB and CTSD activate apoptosis effectors, inducing apoptosis in rat proximal tubular. Cotreatment with CTSB and CTSD inhibitors can significantly inhibit the apoptosis of rat proximal tubular (66). Z-FA-FMK, a highly efficient and specific inhibitor of CTSB, can prevent D-galactosamine/TNF-α-induced apoptosis in renal tubular epithelial cells, further confirming the important role of CTSB in D-galactosamine/TNF-α-induced apoptosis in renal tubular epithelial cells (67, 68). Additionally, CTSB has been utilized as a marker of caspase-independent apoptosis.

Due to the kidney’s high demand for oxygen to maintain metabolic activities, the high oxygen consumption of the active reabsorption function in the renal medulla, and the limited oxygen supply resulting from the renal medulla’s distance from the descending vasa recta, the kidney is particularly sensitive to hypoxic environments (69). Under hypoxia, a large amount of ROS accumulate during metabolic processes. ROS can attack cell membranes and their internal biomolecules, including lipids, proteins, and nucleic acids, leading to cellular dysfunction, triggering apoptosis and inflammatory responses, and further causing acute or chronic kidney injury (70, 71). The connection between ROS and CTSB is complex. The leakage of CTSB from lysosomes contributes to a decrease in the mitochondrial membrane potential, resulting in an increase in mitochondria-derived ROS production (72). Moreover, the ROS generated in mitochondria cause lysosomal membrane lipid peroxidation, which positively promotes LMP via feedback, thereby increasing CTSB activity and expression (73, 74). The Nuclear factor erythroid-2-related factor 2 (NRF2) pathway is a signaling pathway that plays a key role in the cellular defense against oxidative stress and the regulation of antioxidant protein expression. The LVYPFPGPIPN peptide from cattle has been shown to suppress the transcription of inflammatory factors and the apoptosis-related markers CTSB/BAX/Caspase 3 via the NRF2 pathway, thus inhibiting apoptosis and the oxidative stress response and ameliorating hypoxia-induced kidney injury (3, 70, 75).

### 4.2 CTSB induces kidney diseases by mediating pyroptosis

Pyroptosis plays an indispensable role in the development of kidney disease and fibrosis (76). The classic pathway of pyroptosis is mediated by inflammasomes, which can promote the activation of caspase family proteins, especially caspase-1. Cleaved caspase-1 promotes the maturation of interleukin-1β/18 (IL-1β/18) and cleaves gasdermin D (GSDMD). The N-terminal fragment of GSDMD is released and forms a membrane pore, promoting the release of inflammatory factors such as IL-1β and IL-18 into the extracellular space and thereby triggering an inflammatory response and pyroptosis (**Figure 3**) (76–80). Inflammasomes are multimeric protein complexes that can be activated by a variety of substances and signaling molecules. Five main types of inflammasome have been identified, the NOD-like receptor thermal protein domain associated protein 4 (NLRP4), NOD-like receptor thermal protein domain associated protein 3 (NLRP3), NOD-LRR family with CARD 1 (NLRC1), ice-protease activating factor (IPAF), and absent in melanoma 2 (AIM2) inflammasomes, among which the NLRP3 inflammasome plays a central role in kidney diseases. When cells are damaged or invaded by pathogens, damage-associated molecular patterns (DAMPs) and pathogen-associated molecular patterns (PAMPs) are released (81). DAMPs and PAMPs also cause lysosomal disruption and permeabilization by interfering with cellular metabolic pathways or protein degradation pathways. The leakage of CTSB from lysosomes subsequently occurs, and the leaked CTSB is also able to activate the NLRP3 inflammasome, triggering a cascade of inflammatory responses that ultimately lead to pyroptosis (82).

A study of traditional Chinese medicine for the treatment of diabetic nephropathy revealed that ginsenoside Rh2 could ultimately alleviate diabetic nephropathy by inhibiting LMP, reducing the expression of CTSB and cathepsin L, and inhibiting caspase-1-mediated pyroptosis (83, 84). Hyperuricemia has become recognized as an important risk factor for kidney disease. In a hyperuricemia nephropathy (HN) model in which HK-2 cells were exposed to uric acid, the transfection of a CTSB siRNA effectively inhibited the activation of the NLRP3 inflammasome and subsequent pyroptosis (51). Uric acid may activate the NLRP3 inflammasome through the production of ROS in mitochondria, while at the same time, the phagocytosis of urate crystals by macrophages leads to lysosomal rupture and the release of CTSB, which in turn triggers the NLRP3 inflammasome; the two mechanisms mentioned above may work synergistically to promote the development of gouty nephropathy (85, 86). ROS are not only key molecules that activate the NLRP3 inflammasome but also, similar to apoptosis, can activate the NLRP3 inflammasome by increasing CTSB activity and expression (73, 87, 88). When to rat kidney tissue and HK-2 cells are exposed to nephrotoxic concentrations of fluoride, fluoride increases ROS levels, enhances LMP, and promotes the expression of CTSB and CTSD, leading to pyroptosis and inflammation and ultimately causing kidney injury. Rutin reduces the nephrotoxic effects of fluoride by inhibiting the ROS-mediated LMP and activation of the gasdermin E/high mobility group box 1 protein (HMGB1) axis (89).

Renal fibrosis is the result of an inflammatory response and is the product of chronic injury and repair processes. In the event of inflammation, cell damage, or hypoxia, kidney cells release various cytokines and growth factors to promote the proliferation and maturation of fibroblasts, resulting in the proliferation and secretion of collagen and the formation of scar tissue (90). The long-term fibrosis process can lead to impaired renal structural remodeling, ultimately resulting in decreased renal function. Excessive or persistent inflammation has been identified as the core mechanism underlying the development of renal fibrosis (91). CTSB acts as an upstream signaling factor for inflammatory vesicle activation, and the subsequent induction of pyroptosis is a critical factor in the onset of renal fibrosis (91, 92). Hederagenin, a widely distributed pentacyclic triterpenoid compound in various medicinal plants, can inhibit NLRP3 inflammasome activation by reducing CTSB expression and can suppress high glucose-induced fibrosis in human renal mesangial cells and Renal proximal tubular epithelial cells(RPTECs) (93).

### 4.3 CTSB induces kidney diseases by mediating necroptosis

Necroptosis was first discovered in the 1990s and is a regulated form of cellular necrosis. Numerous stimuli have been shown to trigger necroptosis, which primarily involves TNF-α, DNA-dependent activator of interferon-regulatory factors, interferons, and ROS (94, 95). Necroptosis triggered by TNF-α stimulation was the earliest discovered pathway and is the most extensively studied process to date. The biological effects of TNF-α are not limited to triggering necroptosis; it can also trigger apoptosis, which has been discussed in the previous section on TNF-α-induced apoptosis of renal tubular epithelial cells. With the involvement of caspase-8, TNF-α tends to promote apoptosis in cells, which is attributed to the inhibitory effect of caspase-8 on the activity of receptor-interacting protein kinase 1 (RIPK1), a key molecule involved in necroptosis (74). Based on this information, excluding the potential impact of apoptosis in studies of necroptosis is particularly crucial. In the absence of the potential impact of apoptosis, TNF-α binds to its transmembrane receptor tumour necrosis factor receptor 1 (TNFR1), and the activation of TNFR1 promotes the assembly of complex I, which includes TNF receptor-associated death domain, RIPK1, and E3 ubiquitin ligase (96). RIPK1 recruits and activates its homologue RIPK3 through phosphorylation. RIPK3 recruits and phosphorylates the kinase mixed lineage kinase domain-like protein (MLKL), the executioner of the necroptosis pathway (97), which interacts with RIPK3, ultimately disrupting the cell membrane and inducing necroptosis (**Figure 3**).

LMP and the leakage of CTSB are also associated with necroptosis-induced kidney disease (74, 98). In a study of oxalate-induced acute kidney injury (AKI), calcium oxalate (CaOx) crystals were shown to exert direct cytotoxic effects on renal tubular cells by inducing necroptosis associated with the mitochondrial permeability transition (MPT). LMP and CTSB leakage are both involved in the CaOx crystal-induced MPT process. Loss of the mitochondrial outer membrane potential, ROS production, and necrotic apoptosis induced by CaOx crystals are prevented by the CTSB inhibitor CA074Me (99). Similarly, in a diabetic nephropathy model induced by high glucose levels in normal rat kidney-52E cells, CTSB acts as an upstream signal for a decrease in or loss of the mitochondrial membrane potential. It can promote ROS generation. The increased ROS levels can activate RIPK1, recruit RIPK3, promote the formation of the necrosome, and phosphorylate MLKL. Phosphorylated MLKL translocates to the cell membrane and causes membrane permeabilization and rupture, leading to necroptosis in normal rat kidney-52E cells (100); similar results were reported in a cisplatin-induced AKI study (101, 102). Additionally, ROS induce LMP through a positive feedback loop, which further promotes the release of CTSB and CTSD into the cytoplasm, thereby potentiating necroptotic apoptosis (74).

### 4.4 CTSB induces kidney diseases by regulating autophagy

Autophagy is an internal self-digestive process that breaks down and recycles the cell’s own components, such as damaged organelles and proteins, to maintain cellular health and function. Under pathological conditions, autophagy can be abnormally activated, and excessive autophagy or autophagy dysfunction can lead to cell death. Autophagy is intricately linked to several kidney diseases, including acute kidney injury, diabetic nephropathy, and heavy metal-induced kidney injury. However, the occurrence of these kidney injuries is not entirely dependent on autophagic cell death. The damage caused during autophagic clearance often leads to kidney damage by inducing other forms of cell death and activating inflammasomes (103). When human HK-2 cells are exposed to uric acid, autophagy is activated, which further induces the activation of the NLRP3 inflammasome and subsequent pyroptosis, resulting in HN (51). CTSB has been verified to participate in the activation of inflammasomes triggered by autophagy. The increase in autophagy and the degradation of autophagosomes result in the release of CTSB from lysosomes, which in turn activates the NLRP3 inflammasome. The use of autophagy inhibitors can effectively ameliorate kidney damage. 3-methyladenine is an effective inhibitor of autophagy. Treatment of HN rats with 3-methyladenine resulted in a significant decrease in CTSB expression compared to the HN group, and the HN of rats in the 3-methyladenine treatment group improved (51). Under the aforementioned conditions, inhibiting autophagy helps prevent cell death (**Figure 3**).

Current research does not provide direct evidence that CTSB can induce kidney disease by directly mediating autophagic cell death, but CTSB may play a role in kidney disease by regulating autophagy. CTSB is a key protease involved in the degradation of the autophagosomal contents; it controls the expression of autophagy-related proteins and maintains the size and number of autophagosomes in the cell, thereby affecting autophagy flux (104). In kidney diseases, when CTSB is inhibited, autophagy flux decreases, which in turn leads to a decline in renal function. In S-AKI, autophagy has a protective effect on the kidneys. In the LPS-induced S-AKI model, protein kinase 3 impedes the function of transcription factor EB. As a target gene of the transcription factor EB, which is involved in lysosomal function, CTSB expression is also correspondingly downregulated, leading to impaired autophagy and exacerbated renal injury (33). Inhibiting protein kinase 3 can effectively restore autophagy and protect the kidneys. Similarly, Z-VAD-FMK can block the activity of CTSB. In cisplatin-induced kidney injury, Z-VAD-FMK blocks the subsequent maturation of autophagosomes by inhibiting the activity of CTSB, ultimately leading to impaired autophagy flux and a deterioration of kidney function (49). In addition, green tea polyphenols play a similar role in high-fat diet-induced chronic kidney disease, where green tea polyphenols improve autophagy flux by affecting CTSB, which ultimately exerts a protective effect on the kidneys (105).

CTSB is only active in acidic environments. When the pH of lysosomes increases, the activity and expression of acidic hydrolytic enzymes such as CTSB in lysosomes decrease. Sidt2 plays a key role in maintaining lysosomal function and renal physiology. When Sidt2 is absent, lysosomal function is impaired and the internal pH is elevated, leading to a decrease in the activity of hydrolytic enzymes, such as CTSB, which further triggers impaired autophagic degradation and renal dysfunction (106). In studies of kidney injury induced by tacrolimus, tacrolimus damages lysosomal acidification and reduces the activity of CTSB and the expression of the transcription factor EB. Klotho helps alleviate kidney injury by improving the lysosomal dysfunction caused by tacrolimus, thereby reducing the impairment of the autophagy clearance capacity (107).

### 4.5 CTSB induces kidney diseases by mediating ferroptosis

Ferroptosis is a novel form of regulated cell death that is dependent on iron. The depletion of glutathione, inactivation of glutathione peroxidase 4, disruption of iron metabolism, production of oxidative stress, and peroxidation of polyunsaturated fatty acids are characteristic manifestations of ferroptosis (108, 109). Recent studies point to a pivotal role for ferroptosis in the development of various diseases (8, 110). Ferroptosis also plays a key role in kidney diseases such as acute kidney injury, chronic kidney injury and kidney cancer, and inhibiting ferroptosis has been shown to alleviate kidney injury (111, 112). For example, spliceosome-associated protein 130 released by renal tubular epithelial cells undergoing ferroptosis has been shown to promote macrophage polarization via the mincle signaling pathway, which is involved in S-AKI (113). In studies of kidney injury and fibrosis, the synergistic effects of melatonin and zileuton have been shown to inhibit ferroptosis through the protein kinase B/mammalian target of rapamycin/NRF2 signaling pathway (114). In addition, the roles of specific molecules in renal ferroptosis have also been studied. For example, dipeptidase 1 and charged multivesicular body protein 1A have been identified as key factors with roles in renal ferroptosis, suggesting that they regulate this cell death pathway (115). Selenium has also been shown to inhibit the microRNA-202-5p/mitochondrial calcium uptake 1 axis, thereby alleviating renal ferroptosis caused by mercuric chloride (116).

The existing evidence suggests that CTSB mediates ferroptosis and is also referred to by scholars as the executioner of ferroptosis (117–119). Erastin is known to induce ferroptosis, and inhibiting CTSB can reduce the sensitivity of cells to erastin-induced iron deposition, further indicating the involvement of CTSB in the ferroptosis process (120–122). CTSB can be transported from lysosomes to the nucleus, where it can cause nuclear oxidative damage or induce the peroxidation of polyunsaturated fatty acids through the MMP-ROS axis, thereby triggering ferroptosis and the onset of kidney diseases (123, 124). Moreover, erastin can trigger ferroptosis, which is enhanced by the transcription factor EB. Polystyrene nanoparticles trigger lysosomal stress and the nuclear translocation of transcription factor EB, reduce the levels of CTSB, decrease ROS levels, and inhibit ferroptosis (125) (**Figure 3**).

## 5 CTSB as a potential therapeutic target for kidney diseases

The role of CTSB in the occurrence of an increasing number of diseases has been reported, which makes it a hot topic in the research of emerging therapeutic targets. Interventions targeting CTSB, such as inhibiting its activity or regulating its expression levels, have become important strategies for treating various diseases. Thus, CTSB is becoming a new target in disease treatment. In cancer research, CTSB is considered one of the most comprehensive therapeutic targets, and CTSB inhibitors are used as anticancer agents (7, 126, 127). In various diseases, such as inflammatory diseases and neurodegenerative diseases, the inhibition of CTSB helps to slow the progression of the disease (128).

CTSB has also been mentioned many times as an effective intervention target for kidney diseases, including diabetic nephropathy and hypoxia-induced kidney damage (75, 129–131) (**Table 1**). For example, in hypoxia-induced chronic kidney disease, results from network pharmacology and molecular docking research suggest that CTSB and IL-1β may be key targets for treating hypoxic kidney injury (75). In addition, studies on human renal cell carcinoma samples have highlighted the therapeutic potential of CTSB in renal cell carcinoma, and the combined inhibition of the vascular endothelial growth factor and CTSB pathways has become a new treatment strategy for patients with metastatic renal cell carcinoma (47).

**Table 1:** Inhibitors/drugs targeting CTSB or CTSB-related pathway for kidney disease therapy.

Inhibitor/Drugs	CTSB or CTSB-related pathway	Related cells/diseases	Type of cell death	Reference
Metformin	CTSB-ENaC pathway	Collecting duct cells/Diabetes-associated hypertension	/	(45)
Hydroxychloroquine	CTSB	HK-2 cells/ Renal ischemia/reperfusion injury	/	(37)
CA074	CTSB	HK-2 cells/S-AKI	Apoptosis	(65)
CA074	CTSB	Rat proximal tubular/Lead-induced kidney injury	Apoptosis	(66)
Z-FA-FMK	CTSB	Renal tubular epithelial cells/ D-galactosamine or TNF-α-induced kidney injury	Apoptosis	(68)
LVYPFPGPIPN peptide	NRF2 pathway-CTSB	Human embryonic kidney 293 cells/Hypoxia-induced kidney injury	Apoptosis	(75)
Ginsenoside Rh2	LMP-CTSB	Diabetic nephropathy	Pyroptosis	(48)
Rutin	ROS-LMP-CTSB	HK-2 cells/Fluoride-induced kidney injury	Pyroptosis	(89)
Hederagenin	CTSB	Human renal mesangial cells and RPTECs/High glucose-induced kidney fibrosis	Pyroptosis	(93)
CA074Me	CTSB	HK-2 cells and primary renal tubular epithelial cells of mice/Oxalate-induced AKI	Necroptosis	(99)
Z-VAD-FMK	CTSB	Porcine kidney proximal tubule epithelial cells/Cisplatin-induced AKI	Autophagy	(49)
Green tea polyphenols	CTSB	HK-2 cells/High-fat diet-induced chronic kidney disease	Autophagy	(105)
Klotho	LMP-CTSB	HK-2 cells/Tacrolimus-induced renal injury	Autophagy	(107)

In terms of drug development, CTSB is considered a promising target. Although some progress has been made in drug research targeting CTSB, no drugs have been approved for clinical treatment to date. The development and optimization of CTSB inhibitors is an important direction in the field of drug discovery. The inhibitors used in early studies could be classified into three main categories: endogenous inhibitors, synthetic inhibitors, and natural inhibitors (126). Endogenous inhibitors primarily consist of stefins, including stefin A and stefin B, along with cystatin C. Synthetic inhibitors are predominantly CA074 and its derivatives, such as CA074Me. The natural inhibitors used primarily include E-64. Recently, more advanced methods have been researched, such as humanized CTSB antibody inhibitors, nanoparticles that degrade CTSB, intelligent delivery systems that respond to CTSB activity, CTSB-reactive programmed brain-targeted delivery systems, and the development of new dual-functional fluorescent probes for CTSB self-elimination (132–135). In the future, an increasing number of new technologies will be used to target CTSB, opening a new chapter in the treatment of many diseases, including kidney diseases.

## 6 Conclusions

The functions of CTSB, a lysosomal protease, are extensive and complex, and its roles in cellular physiology and pathology cannot be ignored. In the kidney, CTSB plays an irreplaceable role in mediating/regulating the induction of kidney diseases by inducing different types of PCD (3, 136). CTSB may not singularly mediate/regulate a certain PCD-induced renal disease, as described above, during the progression of renal disease. CTSB may be involved in complex renal diseases by mediating/regulating two or more types of PCD. CTSB plays different/similar roles in different forms of PCD, which has potential for the development of new therapies. Future research needs to further explore the interactions between CTSB and different forms of PCD. Kidney diseases, such as AKI and chronic kidney disease, have complex pathological features and lack specific diagnostic and treatment options. CTSB provides scholars with new diagnostic and treatment ideas for kidney diseases. The detection of CTSB may be a method for the early diagnosis of kidney disease, and CTSB-targeted treatment can effectively slow the progression of kidney disease (137).

In conclusion, in-depth research on CTSB not only helps us understand its key roles in physiological and pathological processes but also provides an important direction for the development of new diagnostic and therapeutic strategies. In the future, more functions and the therapeutic potential of CTSB in kidney diseases will be revealed, providing scholars with new strategies and methods and demonstrating its significant value in the field of renal diseases in modern medicine.

## Glossary

AKI: Acute kidney injury
AIM2: Absent in melanoma 2
ASC: Apoptosis-associated speck-like protein containing a CARD
BAX: BCL-2-associated X protein
Bid: BH3 interacting domain death agonist
CaOx: Calcium oxalate
CTSB: Cathepsin B
CTSD: Cathepsin D
DAMPs: Damage-associated molecular patterns
ENaC: Epithelial sodium channels
GSDMD: Gasdermin D
HK-2 cells: Human renal proximal tubular epithelial cell line-2
HMGB1: High mobility group box 1 protein
HN: Hyperuricemia nephropathy
IL-18: Interleukin-18
IL-1β: Interleukin-1β
IPAF: Ice-protease activating factor
LMP: Lysosomal membrane permeabilization
MLKL: Mixed lineage kinase domain-like protein
MMP: Mitochondrial membrane permeabilization
MPT: Mitochondrial permeability transition
N-GSDMD: N-terminal domain of gasdermin D
NLRC1: NOD-LRR family with CARD 1
NLRP3: NOD-like receptor thermal protein domain associated protein 3
NLRP4: NOD-like receptor thermal protein domain associated protein 4
NRF2: Nuclear factor erythroid-2-related factor 2
PAMPs: Pathogen-associated molecular patterns
PCD: Programmed cell death
PKD1: Polycystic kidney disease 1
PRAP: Poly ADP-ribose polymerase
RIPK1: Receptor-interacting protein kinase 1
RIPK3: Receptor-interacting protein kinase 3
ROS: Reactive oxygen species
RPTECs: Renal proximal tubular epithelial cells
S-AKI: Sepsis-induced acute kidney injury
SARS-CoV-2: Severe acute respiratory syndrome coronavirus 2
tBid: Truncated BH3 interacting domain death agonist
TNF-α: Tumour necrosis factor-α
TNFR1: Tumour necrosis factor receptor 1

## References

[1] Lin Z Zhao S Li X Miao Z Cao J Chen Y. Cathepsin B S-nitrosylation promotes ADAR1-mediated editing of its own mRNA transcript via an ADD1/MATR3 regulatory axis. Cell Res. (2023) 33:546–61. doi: 10.1038/s41422-023-00812-4 PMC10313700 37156877

[2] Akinyemi AO Pereira GBS Rocha FV. Role of cathepsin B in cancer progression: A potential target for coordination compounds. Mini Rev Med Chem. (2021) 21:1612–24. doi: 10.2174/1389557521666210212152937 33583372

[3] Xie Z Zhao M Yan C Kong W Lan F Narengaowa. Cathepsin B in programmed cell death machinery: mechanisms of execution and regulatory pathways. Cell Death Dis. (2023) 14:255. doi: 10.1038/s41419-023-05786-0 37031185 PMC10082344

[4] Lim CL Or YZ Ong Z Chung HH Hayashi H Shrestha S. Estrogen exacerbates mammary involution through neutrophil-dependent and -independent mechanism. Elife. (2020) 9:e57274. doi: 10.7554/eLife.57274 32706336 PMC7417171

[5] Aggarwal N Sloane BF. Cathepsin B: multiple roles in cancer. Proteomics Clin Appl. (2014) 8:427–37. doi: 10.1002/prca.201300105 PMC4205946 24677670

[6] Gu Y Zhu Y Deng G Liu S Sun Y Lv W. Curcumin analogue AI-44 alleviates MSU-induced gouty arthritis in mice via inhibiting cathepsin B-mediated NLRP3 inflammasome activation. Int Immunopharmacol. (2021) 93:107375. doi: 10.1016/j.intimp.2021.107375 33517224

[7] Kos J Mitrović A Mirković B. The current stage of cathepsin B inhibitors as potential anticancer agents. Future Med Chem. (2014) 6:1355–71. doi: 10.4155/fmc.14.73 25163003

[8] Lu J Li H Yu Z Cao C Xu Z Peng L. Cathepsin B as a key regulator of ferroptosis in microglia following intracerebral hemorrhage. Neurobiol Dis. (2024) 194:106468. doi: 10.1016/j.nbd.2024.106468 38460801

[9] Ruiz-Blázquez P Pistorio V Fernández-Fernández M Moles A. The multifaceted role of cathepsins in liver disease. J Hepatol. (2021) 75:1192–202. doi: 10.1016/j.jhep.2021.06.031 34242696

[10] Morrone C Smirnova NF Jeridi A Kneidinger N Hollauer C Schupp JC. Cathepsin B promotes collagen biosynthesis, which drives bronchiolitis obliterans syndrome. Eur Respir J. (2021) 57(5). doi: 10.1183/13993003.01416-2020 33303550

[11] Hook G Reinheckel T Ni J Wu Z Kindy M Peters C. Cathepsin B gene knockout improves behavioral deficits and reduces pathology in models of neurologic disorders. Pharmacol Rev. (2022) 74:600–29. doi: 10.1124/pharmrev.121.000527 PMC9553114 35710131

[12] Schmitz J Gilberg E Löser R Bajorath J Bartz U Gütschow M. Cathepsin B: Active site mapping with peptidic substrates and inhibitors. Bioorg Med Chem. (2019) 27:1–15. doi: 10.1016/j.bmc.2018.10.017 30473362

[13] Ni J Lan F Xu Y Nakanishi H Li X. Extralysosomal cathepsin B in central nervous system: Mechanisms and therapeutic implications. Brain Pathol. (2022) 32:e13071. doi: 10.1111/bpa.13071 35411983 PMC9425006

[14] Cai Z Xu S Liu C. Cathepsin B in cardiovascular disease: Underlying mechanisms and therapeutic strategies. J Cell Mol Med. (2024) 28:e70064. doi: 10.1111/jcmm.70064 39248527 PMC11382359

[15] Otomo T Schweizer M Kollmann K Schumacher V Muschol N Tolosa E. Mannose 6 phosphorylation of lysosomal enzymes controls B cell functions. J Cell Biol. (2015) 208:171–80. doi: 10.1083/jcb.201407077 PMC4298682 25601403

[16] Gonzalez-Leal IJ Röger B Schwarz A Schirmeister T Reinheckel T Lutz MB. Cathepsin B in antigen-presenting cells controls mediators of the Th1 immune response during Leishmania major infection. PloS Negl Trop Dis. (2014) 8:e3194. doi: 10.1371/journal.pntd.0003194 25255101 PMC4177854

[17] Gorgoulis V Adams PD Alimonti A Bennett DC Bischof O Bishop C. Cellular senescence: defining a path forward. Cell. (2019) 179:813–27. doi: 10.1016/j.cell.2019.10.005 31675495

[18] Yokota S Tsuji H Kato K. Immunocytochemical localization of cathepsin B in rat kidney. I. Light microscopic study using the indirect immunoenzyme technique. J Histochem Cytochem. (1986) 34:891–7. doi: 10.1177/34.7.3519752 3519752

[19] Saudenova M Promnitz J Ohrenschall G Himmerkus N Böttner M Kunke M. Behind every smile there's teeth: Cathepsin B's function in health and disease with a kidney view. Biochim Biophys Acta Mol Cell Res. (2022) 1869:119190. doi: 10.1016/j.bbamcr.2021.119190 34968578

[20] Yokota S Kato K. Involvement of cathepsins B and H in lysosomal degradation of horseradish peroxidase endocytosed by the proximal tubule cells of the rat kidney: II. Immunocytochemical studies using protein A-gold technique applied to conventional and serial sections. Anat Rec. (1988) 221:791–801. doi: 10.1002/ar.1092210403 3056113

[21] Daehn IS Duffield JS. The glomerular filtration barrier: a structural target for novel kidney therapies. Nat Rev Drug Discovery. (2021) 20:770–88. doi: 10.1038/s41573-021-00242-0 PMC8278373 34262140

[22] Zheng F Tang D Li S Luo Z Song Y Huang Y. Spatial proteomics landscape and immune signature analysis of renal sample of lupus nephritis based on laser-captured microsection. Inflammation Res. (2023) 72:1603–20. doi: 10.1007/s00011-023-01767-3 PMC10499763 37474625

[23] Baricos WH Cortez SL Le QC Zhou YW Dicarlo RM O'connor SE. Glomerular basement membrane degradation by endogenous cysteine proteinases in isolated rat glomeruli. Kidney Int. (1990) 38:395–401. doi: 10.1038/ki.1990.218 2232482

[24] Davies M Hughes KT Thomas GJ. Evidence that kidney lysosomal proteinases degrade the collagen of glomerular basement membrane. Ren Physiol. (1980) 3:116–9. doi: 10.1159/000172750 7034089

[25] Leehey DJ Song RH Alavi N Singh AK. Decreased degradative enzymes in mesangial cells cultured in high glucose media. Diabetes. (1995) 44:929–35. doi: 10.2337/diab.44.8.929 7621999

[26] Salem RM Todd JN Sandholm N Cole JB Chen WM Andrews D. Genome-wide association study of diabetic kidney disease highlights biology involved in glomerular basement membrane collagen. J Am Soc Nephrol. (2019) 30:2000–16. doi: 10.1681/asn.2019030218 PMC6779358 31537649

[27] Blaine J Dylewski J. Regulation of the actin cytoskeleton in podocytes. Cells. (2020) 9(7). doi: 10.3390/cells9071700 PMC7408282 32708597

[28] Wu M Zhang M Zhang Y Li Z Li X Liu Z. Relationship between lysosomal dyshomeostasis and progression of diabetic kidney disease. Cell Death Dis. (2021) 12:958. doi: 10.1038/s41419-021-04271-w 34663802 PMC8523726

[29] Liu WJ Gan Y Huang WF Wu HL Zhang XQ Zheng HJ. Lysosome restoration to activate podocyte autophagy: a new therapeutic strategy for diabetic kidney disease. Cell Death Dis. (2019) 10:806. doi: 10.1038/s41419-019-2002-6 31649253 PMC6813305

[30] Bi L Hou R Yang D Li S Zhao D. Erythropoietin protects lipopolysaccharide-induced renal mesangial cells from autophagy. Exp Ther Med. (2015) 9:559–62. doi: 10.3892/etm.2014.2124 PMC4280982 25574234

[31] Xu L Fan Q Wang X Zhao X Wang L. Inhibition of autophagy increased AGE/ROS-mediated apoptosis in mesangial cells. Cell Death Dis. (2016) 7:e2445. doi: 10.1038/cddis.2016.322 27809300 PMC5260901

[32] Höhne M Frese CK Grahammer F Dafinger C Ciarimboli G Butt L. Single-nephron proteomes connect morphology and function in proteinuric kidney disease. Kidney Int. (2018) 93:1308–19. doi: 10.1016/j.kint.2017.12.012 29530281

[33] Li R Zhao X Zhang S Dong W Zhang L Chen Y. RIP3 impedes transcription factor EB to suppress autophagic degradation in septic acute kidney injury. Cell Death Dis. (2021) 12:593. doi: 10.1038/s41419-021-03865-8 34103472 PMC8187512

[34] D'agati VD Chagnac A De Vries AP Levi M Porrini E Herman-Edelstein M. Obesity-related glomerulopathy: clinical and pathologic characteristics and pathogenesis. Nat Rev Nephrol. (2016) 12:453–71. doi: 10.1038/nrneph.2016.75 27263398

[35] Lin H Ma X Xiao F Su H Shi Y Liu Y. Identification of a special cell type as a determinant of the kidney tropism of SARS-CoV-2. FEBS J. (2021) 288:5163–78. doi: 10.1111/febs.16114 PMC8420455 34228902

[36] Vanslambrouck JM Neil JA Rudraraju R Mah S Tan KS Groenewegen E. Kidney organoids reveal redundancy in viral entry pathways during ACE2-dependent SARS-CoV-2 infection. J Virol. (2024) 98:e0180223. doi: 10.1128/jvi.01802-23 38334329 PMC10949421

[37] Tang TT Lv LL Pan MM Wen Y Wang B Li ZL. Hydroxychloroquine attenuates renal ischemia/reperfusion injury by inhibiting cathepsin mediated NLRP3 inflammasome activation. Cell Death Dis. (2018) 9:351. doi: 10.1038/s41419-018-0378-3 29500339 PMC5834539

[38] Zhang X Zhou Y Yu X Huang Q Fang W Li J. Differential roles of cysteinyl cathepsins in TGF-β Signaling and tissue fibrosis. iScience. (2019) 19:607–22. doi: 10.1016/j.isci.2019.08.014 PMC6715892 31446224

[39] Peintner L Venkatraman A Waeldin A Hofherr A Busch T Voronov A. Loss of PKD1/polycystin-1 impairs lysosomal activity in a CAPN (calpain)-dependent manner. Autophagy. (2021) 17:2384–400. doi: 10.1080/15548627.2020.1826716 PMC8496716 32967521

[40] Gholam MF Bala N Dogan YE Alli AA. Augmentation of cathepsin isoforms in diabetic db/db mouse kidneys is associated with an increase in renal MARCKS expression and proteolysis. Int J Mol Sci. (2023) 24(15). doi: 10.3390/ijms241512484 PMC10419664 37569859

[41] Kawakibi T Bala N Liu LP Searcy LA Denslow ND Alli AA. Decreased MARCKS protein expression in kidney cortex membrane fractions of cathepsin B knockout mice is associated with reduced lysophosphatidylcholine and protein kinase C activity. Biomedicines. (2023) 11(5). doi: 10.3390/biomedicines11051489 PMC10216610 37239160

[42] Alli AA Song JZ Al-Khalili O Bao HF Ma HP Alli AA. Cathepsin B is secreted apically from Xenopus 2F3 cells and cleaves the epithelial sodium channel (ENaC) to increase its activity. J Biol Chem. (2012) 287:30073–83. doi: 10.1074/jbc.M111.338574 PMC3436264 22782900

[43] Ha SD Martins A Khazaie K Han J Chan BM Kim SO. Cathepsin B is involved in the trafficking of TNF-alpha-containing vesicles to the plasma membrane in macrophages. J Immunol. (2008) 181:690–7. doi: 10.4049/jimmunol.181.1.690 18566436

[44] Larionov A Dahlke E Kunke M Zanon Rodriguez L Schiessl IM Magnin JL. Cathepsin B increases ENaC activity leading to hypertension early in nephrotic syndrome. J Cell Mol Med. (2019) 23:6543–53. doi: 10.1111/jcmm.14387 PMC6787568 31368174

[45] ScIndia YM Gholam MF Waleed A Liu LP Chacko KM Desai D. Metformin alleviates diabetes-associated hypertension by attenuating the renal epithelial sodium channel. Biomedicines. (2023) 11(2). doi: 10.3390/biomedicines11020305 PMC9953274 36830842

[46] Baricos WH Shah SV. Role of cathepsin B and L in anti-glomerular basement membrane nephritis in rats. Ren Physiol Biochem. (1989) 12:400–5. doi: 10.1159/000173218 2623352

[47] Rudzinska-Radecka M Frolova AS Balakireva AV Gorokhovets NV Pokrovsky VS Sokolova DV. In silico, *in vitro*, and clinical investigations of cathepsin B and stefin A mRNA expression and a correlation analysis in kidney cancer. Cells. (2022) 11(9). doi: 10.3390/cells11091455 PMC9101197 35563761

[48] Zhao W He C Wang F. Screening potential Chinese materia medica and their monomers for treatment diabetic nephropathy based on caspase-1-mediated pyroptosis. Nan Fang Yi Ke Da Xue Xue Bao. (2020) 40:1280–7. doi: 10.12122/j.issn.1673-4254.2020.09.09 PMC7544587 32990240

[49] Herzog C Yang C Holmes A Kaushal GP. zVAD-fmk prevents cisplatin-induced cleavage of autophagy proteins but impairs autophagic flux and worsens renal function. Am J Physiol Renal Physiol. (2012) 303:F1239–50. doi: 10.1152/ajprenal.00659.2011 PMC3469677 22896037

[50] Chen CH Bhasin S Khanna P Joshi M Joslin PM Saxena R. Study of Cathepsin B inhibition in VEGFR TKI treated human renal cell carcinoma xenografts. Oncogenesis. (2019) 8:15. doi: 10.1038/s41389-019-0121-7 30796200 PMC6386754

[51] Hu Y Shi Y Chen H Tao M Zhou X Li J. Blockade of autophagy prevents the progression of hyperuricemic nephropathy through inhibiting NLRP3 inflammasome-mediated pyroptosis. Front Immunol. (2022) 13:858494. doi: 10.3389/fimmu.2022.858494 35309342 PMC8924517

[52] Shillingford JM Shayman JA. Functional TFEB activation characterizes multiple models of renal cystic disease and loss of polycystin-1. Am J Physiol Renal Physiol. (2023) 324:F404–f22. doi: 10.1152/ajprenal.00237.2022 PMC10069964 36794754

[53] Wang M Yu F Zhang Y Li P. Programmed cell death in tumor immunity: mechanistic insights and clinical implications. Front Immunol. (2023) 14:1309635. doi: 10.3389/fimmu.2023.1309635 38283351 PMC10811021

[54] Yuan J Ofengeim D. A guide to cell death pathways. Nat Rev Mol Cell Biol. (2024) 25:379–95. doi: 10.1038/s41580-023-00689-6 38110635

[55] Kari S Subramanian K Altomonte IA Murugesan A Yli-Harja O Kandhavelu M. Programmed cell death detection methods: a systematic review and a categorical comparison. Apoptosis. (2022) 27:482–508. doi: 10.1007/s10495-022-01735-y 35713779 PMC9308588

[56] Reinheckel T Tholen M. Low-level lysosomal membrane permeabilization for limited release and sublethal functions of cathepsin proteases in the cytosol and nucleus. FEBS Open Bio. (2022) 12:694–707. doi: 10.1002/2211-5463.13385 PMC8972055 35203107

[57] Serrano-Puebla A Boya P. Lysosomal membrane permeabilization in cell death: new evidence and implications for health and disease. Ann N Y Acad Sci. (2016) 1371:30–44. doi: 10.1111/nyas.12966 26599521

[58] Chapman HA. Cathepsins as transcriptional activators? Dev Cell. (2004) 6:610–1. doi: 10.1016/s1534-5807(04)00141-8 15130484

[59] Stahl-Meyer J Stahl-Meyer K Jäättelä M. Control of mitosis, inflammation, and cell motility by limited leakage of lysosomes. Curr Opin Cell Biol. (2021) 71:29–37. doi: 10.1016/j.ceb.2021.02.003 33684809

[60] Wang F Gómez-Sintes R Boya P. Lysosomal membrane permeabilization and cell death. Traffic. (2018) 19:918–31. doi: 10.1111/tra.12613 30125440

[61] Liu WJ Xu BH Ye L Liang D Wu HL Zheng YY. Urinary proteins induce lysosomal membrane permeabilization and lysosomal dysfunction in renal tubular epithelial cells. Am J Physiol Renal Physiol. (2015) 308:F639–49. doi: 10.1152/ajprenal.00383.2014 25587119

[62] Luke CJ Markovina S Good M Wight IE Thomas BJ Linneman JM. Lysoptosis is an evolutionarily conserved cell death pathway moderated by intracellular serpins. Commun Biol. (2022) 5:47. doi: 10.1038/s42003-021-02953-x 35022507 PMC8755814

[63] Makhammajanov Z Gaipov A Myngbay A Bukasov R Aljofan M Kanbay M. Tubular toxicity of proteinuria and the progression of chronic kidney disease. Nephrol Dial Transplant. (2024) 39:589–99. doi: 10.1093/ndt/gfad215 37791392

[64] Shamekhi Amiri F. Intracellular organelles in health and kidney disease. Nephrol Ther. (2019) 15:9–21. doi: 10.1016/j.nephro.2018.04.002 29887266

[65] Wang Y Xi W Zhang X Bi X Liu B Zheng X. CTSB promotes sepsis-induced acute kidney injury through activating mitochondrial apoptosis pathway. Front Immunol. (2022) 13:1053754. doi: 10.3389/fimmu.2022.1053754 36713420 PMC9880165

[66] Song XB Liu G Liu F Yan ZG Wang ZY Liu ZP. Autophagy blockade and lysosomal membrane permeabilization contribute to lead-induced nephrotoxicity in primary rat proximal tubular cells. Cell Death Dis. (2017) 8:e2863. doi: 10.1038/cddis.2017.262 28594408 PMC5520918

[67] Singhal PC Franki N Kumari S Sanwal V Wagner JD Mattana J. Extracellular matrix modulates mesangial cell apoptosis and mRNA expression of cathepsin-B and tissue transglutaminase. J Cell Biochem. (1998) 68:22–30. doi: 10.1002/(sici)1097-4644(19980101)68:1<22::aid-jcb3>3.0.co;2-y 9407311

[68] Gezginci-Oktayoglu S Tunali S Yanardag R Bolkent S. Effects of Z-FA.FMK on D-galactosamine/tumor necrosis factor-alpha-induced kidney injury and oxidative stress in mice: effects of Z-FA.FMK on TNF-alpha-mediated kidney injury. Mol Cell Biochem. (2008) 309:9–20. doi: 10.1007/s11010-007-9636-x 18008146

[69] Vio CP Salas D Cespedes C Diaz-Elizondo J Mendez N Alcayaga J. Imbalance in renal vasoactive enzymes induced by mild hypoxia: angiotensin-converting enzyme increases while neutral endopeptidase decreases. Front Physiol. (2018) 9:1791. doi: 10.3389/fphys.2018.01791 30618804 PMC6297360

[70] Voronina MV Frolova AS Kolesova EP Kuldyushev NA Parodi A Zamyatnin AA Jr. The intricate balance between life and death: ROS, cathepsins, and their interplay in cell death and autophagy. Int J Mol Sci. (2024) 25(7). doi: 10.3390/ijms25074087 PMC11012956 38612897

[71] Wang B Li ZL Zhang YL Wen Y Gao YM Liu BC. Hypoxia and chronic kidney disease. EBioMedicine. (2022) 77:103942. doi: 10.1016/j.ebiom.2022.103942 35290825 PMC8921539

[72] Ni J Wu Z Stoka V Meng J Hayashi Y Peters C. Increased expression and altered subcellular distribution of cathepsin B in microglia induce cognitive impairment through oxidative stress and inflammatory response in mice. Aging Cell. (2019) 18:e12856. doi: 10.1111/acel.12856 30575263 PMC6351837

[73] Bai H Yang B Yu W Xiao Y Yu D Zhang Q. Cathepsin B links oxidative stress to the activation of NLRP3 inflammasome. Exp Cell Res. (2018) 362:180–7. doi: 10.1016/j.yexcr.2017.11.015 29196167

[74] Alu A Han X Ma X Wu M Wei Y Wei X. The role of lysosome in regulated necrosis. Acta Pharm Sin B. (2020) 10:1880–903. doi: 10.1016/j.apsb.2020.07.003 PMC7606114 33163342

[75] Yang F Chu Z Wu Q Qu G He Z An J. A peptide from yak ameliorates hypoxia-induced kidney injury by inhibiting inflammation and apoptosis via Nrf2 pathway. Food Bioscience. (2024) 60:104407. doi: 10.1016/j.fbio.2024.104407

[76] Elias EE Lyons B Muruve DA. Gasdermins and pyroptosis in the kidney. Nat Rev Nephrol. (2023) 19:337–50. doi: 10.1038/s41581-022-00662-0 36596918

[77] Mehrotra P Maschalidi S Boeckaerts L Maueröder C Tixeira R Pinney J. Oxylipins and metabolites from pyroptotic cells act as promoters of tissue repair. Nature. (2024) 631:207–15. doi: 10.1038/s41586-024-07585-9 38926576

[78] Rao Z Zhu Y Yang P Chen Z Xia Y Qiao C. Pyroptosis in inflammatory diseases and cancer. Theranostics. (2022) 12:4310–29. doi: 10.7150/thno.71086 PMC9169370 35673561

[79] Coll RC Schroder K Pelegrín P. NLRP3 and pyroptosis blockers for treating inflammatory diseases. Trends Pharmacol Sci. (2022) 43:653–68. doi: 10.1016/j.tips.2022.04.003 35513901

[80] Burdette BE Esparza AN Zhu H Wang S. Gasdermin D in pyroptosis. Acta Pharm Sin B. (2021) 11:2768–82. doi: 10.1016/j.apsb.2021.02.006 PMC8463274 34589396

[81] Schroder K Tschopp J. The inflammasomes. Cell. (2010) 140:821–32. doi: 10.1016/j.cell.2010.01.040 20303873

[82] Campden RI Zhang Y. The role of lysosomal cysteine cathepsins in NLRP3 inflammasome activation. Arch Biochem Biophys. (2019) 670:32–42. doi: 10.1016/j.abb.2019.02.015 30807742

[83] Mamun AA Wu Y Nasrin F Akter A Taniya MA Munir F. Role of pyroptosis in diabetes and its therapeutic implications. J Inflammation Res. (2021) 14:2187–206. doi: 10.2147/jir.S291453 PMC8164340 34079327

[84] Cao Z Huang D Tang C Lu Y Huang S Peng C. Pyroptosis in diabetes and diabetic nephropathy. Clin Chim Acta. (2022) 531:188–96. doi: 10.1016/j.cca.2022.04.011 35427562

[85] Shi X Zhuang L Zhai Z He Y Sun E. Polydatin protects against gouty nephropathy by inhibiting renal tubular cell pyroptosis. Int J Rheum Dis. (2023) 26:116–23. doi: 10.1111/1756-185x.14463 36328529

[86] Ahn H Lee G Lee GS. Lower temperatures exacerbate NLRP3 inflammasome activation by promoting monosodium urate crystallization, causing gout. Cells. (2021) 10(8). doi: 10.3390/cells10081919 PMC8394355 34440688

[87] Swanson KV Deng M Ting JP. The NLRP3 inflammasome: molecular activation and regulation to therapeutics. Nat Rev Immunol. (2019) 19:477–89. doi: 10.1038/s41577-019-0165-0 PMC7807242 31036962

[88] Fusco R Siracusa R Genovese T Cuzzocrea S Di Paola R. Focus on the role of NLRP3 inflammasome in diseases. Int J Mol Sci. (2020) 21(12). doi: 10.3390/ijms21124223 PMC7352196 32545788

[89] Ma Y Xu P Xing H Zhang Y Li T Ding X. Rutin mitigates fluoride-induced nephrotoxicity by inhibiting ROS-mediated lysosomal membrane permeabilization and the GSDME-HMGB1 axis involved in pyroptosis and inflammation. Ecotoxicol Environ Saf. (2024) 274:116195. doi: 10.1016/j.ecoenv.2024.116195 38479315

[90] Humphreys BD. Mechanisms of renal fibrosis. Annu Rev Physiol. (2018) 80:309–26. doi: 10.1146/annurev-physiol-022516-034227 29068765

[91] Liu Y Lei H Zhang W Xing Q Liu R Wu S. Pyroptosis in renal inflammation and fibrosis: current knowledge and clinical significance. Cell Death Dis. (2023) 14:472. doi: 10.1038/s41419-023-06005-6 37500614 PMC10374588

[92] Song Z Gong Q Guo J. Pyroptosis: mechanisms and links with fibrosis. Cells. (2021) 10(12). doi: 10.3390/cells10123509 PMC8700428 34944017

[93] Yang G Yang W Jiang H Yi Q Ma W. Hederagenin inhibits high glucose-induced fibrosis in human renal cells by suppression of NLRP3 inflammasome activation through reducing cathepsin B expression. Chem Biol Drug Des. (2023) 102:1409–20. doi: 10.1111/cbdd.14332 37599208

[94] Linkermann A Green DR. Necroptosis. N Engl J Med. (2014) 370:455–65. doi: 10.1056/NEJMra1310050 PMC4035222 24476434

[95] Pasparakis M Vandenabeele P. Necroptosis and its role in inflammation. Nature. (2015) 517:311–20. doi: 10.1038/nature14191 25592536

[96] Du J Xiang Y Liu H Liu S Kumar A Xing C. RIPK1 dephosphorylation and kinase activation by PPP1R3G/PP1γ promote apoptosis and necroptosis. Nat Commun. (2021) 12:7067. doi: 10.1038/s41467-021-27367-5 34862394 PMC8642546

[97] Grootjans S Vanden Berghe T Vandenabeele P. Initiation and execution mechanisms of necroptosis: an overview. Cell Death Differ. (2017) 24:1184–95. doi: 10.1038/cdd.2017.65 PMC5520172 28498367

[98] Chen H Fang Y Wu J Chen H Zou Z Zhang X. RIPK3-MLKL-mediated necroinflammation contributes to AKI progression to CKD. Cell Death Dis. (2018) 9:878. doi: 10.1038/s41419-018-0936-8 30158627 PMC6115414

[99] Mulay SR Honarpisheh MM Foresto-Neto O Shi C Desai J Zhao ZB. Mitochondria permeability transition versus necroptosis in oxalate-induced AKI. J Am Soc Nephrol. (2019) 30:1857–69. doi: 10.1681/asn.2018121218 PMC6779355 31296606

[100] Guo M Chen Q Huang Y Wu Q Zeng Y Tan X. High glucose-induced kidney injury via activation of necroptosis in diabetic kidney disease. Oxid Med Cell Longev. (2023) 2023:2713864. doi: 10.1155/2023/2713864 36756299 PMC9902134

[101] Alassaf N Attia H. Autophagy and necroptosis in cisplatin-induced acute kidney injury: Recent advances regarding their role and therapeutic potential. Front Pharmacol. (2023) 14:1103062. doi: 10.3389/fphar.2023.1103062 36794281 PMC9922871

[102] Al-Salam S Jagadeesh GS Sudhadevi M Tageldeen H Yasin J. Galectin-3 possesses anti-necroptotic and anti-apoptotic effects in cisplatin-induced acute tubular necrosis. Cell Physiol Biochem. (2021) 55:344–63. doi: 10.33594/000000381 34171186

[103] Chen XC Li ZH Yang C Tang JX Lan HY Liu HF. Lysosome depletion-triggered autophagy impairment in progressive kidney injury. Kidney Dis (Basel). (2021) 7:254–67. doi: 10.1159/000515035 PMC8314772 34395541

[104] Man SM Kanneganti TD. Regulation of lysosomal dynamics and autophagy by CTSB/cathepsin B. Autophagy. (2016) 12:2504–5. doi: 10.1080/15548627.2016.1239679 PMC5173259 27786577

[105] Xie X Yi W Zhang P Wu N Yan Q Yang H. Green tea polyphenols, mimicking the effects of dietary restriction, ameliorate high-fat diet-induced kidney injury via regulating autophagy flux. Nutrients. (2017) 9:497. doi: 10.3390/nu9050497 28505110 PMC5452227

[106] Geng MY Wang L Song YY Gu J Hu X Yuan C. Sidt2 is a key protein in the autophagy-lysosomal degradation pathway and is essential for the maintenance of kidney structure and filtration function. Cell Death Dis. (2021) 13:7. doi: 10.1038/s41419-021-04453-6 34923568 PMC8684554

[107] Lim SW Shin YJ Luo K Quan Y Ko EJ Chung BH. Effect of Klotho on autophagy clearance in tacrolimus-induced renal injury. FASEB J. (2019) 33:2694–706. doi: 10.1096/fj.201800751R 30307767

[108] Fratta Pasini AM Stranieri C Girelli D Busti F Cominacini L. Is ferroptosis a key component of the process leading to multiorgan damage in COVID-19? Antioxidants (Basel). (2021) 10(11). doi: 10.3390/antiox10111677 PMC8615234 34829548

[109] Bayır H Dixon SJ Tyurina YY Kellum JA Kagan VE. Ferroptotic mechanisms and therapeutic targeting of iron metabolism and lipid peroxidation in the kidney. Nat Rev Nephrol. (2023) 19:315–36. doi: 10.1038/s41581-023-00689-x 36922653

[110] Luo L Chen H Xie K Xiang J Chen J Lin Z. Cathepsin B serves as a potential prognostic biomarker and correlates with ferroptosis in rheumatoid arthritis. Int Immunopharmacol. (2024) 128:111502. doi: 10.1016/j.intimp.2024.111502 38199197

[111] Qin J Li Z Feng Y Guo Y Zhao Z Sun S. Reactive oxygen species-scavenging mesoporous poly(tannic acid) nanospheres alleviate acute kidney injury by inhibiting ferroptosis. ACS Biomater Sci Eng. (2024) 10:5856–68. doi: 10.1021/acsbiomaterials.4c00844 PMC11389690 39164198

[112] Yang L Liu Y Zhou S Feng Q Lu Y Liu D. Novel insight into ferroptosis in kidney diseases. Am J Nephrol. (2023) 54:184–99. doi: 10.1159/000530882 37231767

[113] Zhang J Jiang J Wang B Wang Y Qian Y Suo J. SAP130 released by ferroptosis tubular epithelial cells promotes macrophage polarization via Mincle signaling in sepsis acute kidney injury. Int Immunopharmacol. (2024) 129:111564. doi: 10.1016/j.intimp.2024.111564 38320352

[114] Jung KH Kim SE Go HG Lee YJ Park MS Ko S. Synergistic renoprotective effect of melatonin and zileuton by inhibition of ferroptosis via the AKT/mTOR/NRF2 signaling in kidney injury and fibrosis. Biomol Ther (Seoul). (2023) 31:599–610. doi: 10.4062/biomolther.2023.062 37183002 PMC10616517

[115] Allison SJ. DPEP1 and CHMP1A in kidney ferroptosis. Nat Rev Nephrol. (2021) 17:707. doi: 10.1038/s41581-021-00496-2 34561671

[116] Li Y Cui H Xu WX Fu HY Li JZ Fan RF. Selenium represses microRNA-202-5p/MICU1 aixs to attenuate mercuric chloride-induced kidney ferroptosis. Poult Sci. (2024) 103:103891. doi: 10.1016/j.psj.2024.103891 38878746 PMC11227010

[117] Nagakannan P Islam MI Conrad M Eftekharpour E. Cathepsin B is an executioner of ferroptosis. Biochim Biophys Acta Mol Cell Res. (2021) 1868:118928. doi: 10.1016/j.bbamcr.2020.118928 33340545

[118] Liu K Liu J Zou B Li C Zeh HJ Kang R. Trypsin-mediated sensitization to ferroptosis increases the severity of pancreatitis in mice. Cell Mol Gastroenterol Hepatol. (2022) 13:483–500. doi: 10.1016/j.jcmgh.2021.09.008 34562639 PMC8688567

[119] Kuang F Liu J Li C Kang R Tang D. Cathepsin B is a mediator of organelle-specific initiation of ferroptosis. Biochem Biophys Res Commun. (2020) 533:1464–9. doi: 10.1016/j.bbrc.2020.10.035 33268027

[120] Tang D Chen X Kang R Kroemer G. Ferroptosis: molecular mechanisms and health implications. Cell Res. (2021) 31:107–25. doi: 10.1038/s41422-020-00441-1 PMC8026611 33268902

[121] Liu J Chen J Lv J Gong Y Song J. The mechanisms of ferroptosis in the pathogenesis of kidney diseases. J Nephrol. (2024) 37:865–79. doi: 10.1007/s40620-024-01927-6 38704472

[122] Chen X Yu C Kang R Kroemer G Tang D. Cellular degradation systems in ferroptosis. Cell Death Differ. (2021) 28:1135–48. doi: 10.1038/s41418-020-00728-1 PMC8027807 33462411

[123] Chen X Kang R Kroemer G Tang D. Organelle-specific regulation of ferroptosis. Cell Death Differ. (2021) 28:2843–56. doi: 10.1038/s41418-021-00859-z PMC8481335 34465893

[124] Liu J Kang R Tang D. Signaling pathways and defense mechanisms of ferroptosis. FEBS J. (2022) 289:7038–50. doi: 10.1111/febs.16059 34092035

[125] Li L Sun S Tan L Wang Y Wang L Zhang Z. Polystyrene nanoparticles reduced ROS and inhibited ferroptosis by triggering lysosome stress and TFEB nucleus translocation in a size-dependent manner. Nano Lett. (2019) 19:7781–92. doi: 10.1021/acs.nanolett.9b02795 31558022

[126] Liu F Zhou T Zhang S Li Y Chen Y Miao Z. Cathepsin B: The dawn of tumor therapy. Eur J Med Chem. (2024) 269:116329. doi: 10.1016/j.ejmech.2024.116329 38508117

[127] Zamyatnin AA Jr. Gregory LC Townsend PA Soond SM. Beyond basic research: the contribution of cathepsin B to cancer development, diagnosis and therapy. Expert Opin Ther Targets. (2022) 26:963–77. doi: 10.1080/14728222.2022.2161888 36562407

[128] Hook G Jacobsen JS Grabstein K Kindy M Hook V. Cathepsin B is a new drug target for traumatic brain injury therapeutics: evidence for E64d as a promising lead drug candidate. Front Neurol. (2015) 6:178. doi: 10.3389/fneur.2015.00178 26388830 PMC4557097

[129] Wyczalkowska-Tomasik A Bartlomiejczyk I Wirkowska A Koperski L Gornicka B Paczek L. The blocking on the cathepsin B and fibronectin accumulation in kidney glomeruli of diabetic rats. Int J Endocrinol. (2015) 2015:812825. doi: 10.1155/2015/812825 26089895 PMC4452094

[130] Muniyappa R Sowers JR. Glycogen synthase kinase-3β and cathepsin B in diabetic endothelial progenitor cell dysfunction: an old player finds a new partner. Diabetes. (2014) 63:1194–7. doi: 10.2337/db14-0004 PMC3964509 24651804

[131] Kumar Vr S Anders HJ. Cathepsins are potential therapeutic targets in kidney disease. Kidney Int. (2016) 90:933–5. doi: 10.1016/j.kint.2016.07.034 27742196

[132] Jiang S Li W Yang J Zhang T Zhang Y Xu L. Cathepsin B-responsive programmed brain targeted delivery system for chemo-immunotherapy combination therapy of glioblastoma. ACS Nano. (2024) 18:6445–62. doi: 10.1021/acsnano.3c11958 38358804

[133] Egorova VS Kolesova EP Lopus M Yan N Parodi A Zamyatnin AA Jr. Smart delivery systems responsive to cathepsin B activity for cancer treatment. Pharmaceutics. (2023) 15(7). doi: 10.3390/pharmaceutics15071848 PMC10386206 37514035

[134] Li Y Xia X Niu Z Wang K Liu J Li X. _h_CeO_2_@ Cu_5.4_O nanoparticle alleviates inflammatory responses by regulating the CTSB-NLRP3 signaling pathway. Front Immunol. (2024) 15:1344098. doi: 10.3389/fimmu.2024.1344098 38711511 PMC11070469

[135] Dai Z Cheng Q Zhang Y. Rational design of a humanized antibody inhibitor of cathepsin B. Biochemistry. (2020) 59:1420–7. doi: 10.1021/acs.biochem.0c00046 PMC7184884 32212642

[136] Wang Y Li Y Xu Y. Pyroptosis in kidney disease. J Mol Biol. (2022) 434:167290. doi: 10.1016/j.jmb.2021.167290 34626644

[137] Wang C Du W Wu C Dan S Sun M Zhang T. Cathespin B-initiated cypate nanoparticle formation for tumor photoacoustic imaging. Angew Chem Int Ed Engl. (2022) 61:e202114766. doi: 10.1002/anie.202114766 34878207

